# Functionalized bioadhesion-enhanced carboxymethyl cellulose/polyvinyl alcohol hybrid hydrogels for chronic wound dressing applications†

**DOI:** 10.1039/d3ra01519j

**Published:** 2023-04-28

**Authors:** A. A. P. Mansur, M. A. Rodrigues, N. S. V. Capanema, S. M. Carvalho, D. A. Gomes, H. S. Mansur

**Affiliations:** a Department of Metallurgical and Materials Engineering, Center of Nanoscience, Nanotechnology and Innovation – CeNano^2^I, Federal University of Minas Gerais Av. Antônio Carlos 6627 – Escola de Engenharia, Bloco 2 – Sala 2233, 31.270-901 Belo Horizonte MG Brazil hmansur@demet.ufmg.br +55-31-34091843; b Department of Biochemistry and Immunology, Federal University of Minas Gerais Brazil

## Abstract

Wounds produced by trauma, burns, and chronic diseases cause millions of patients to suffer discomfort, pain, and, in many cases, disability and death, leading to enormous health, social and financial impacts globally. Regrettably, current clinical treatments for chronic wounds remain unsatisfactory. Thus, this study reports for the first time the design, development, and synthesis of chemically biofunctionalized hybrid hydrogels made of carboxymethyl cellulose (CMC) and poly(vinyl alcohol) (PVA) crosslinked by citric acid using an entirely biocompatible and green process. They demonstrated suitable physicochemical properties, cytocompatibility, and hemocompatibility to be applied as a smart wound dressing for skin tissue engineering. These novel hybrids were biofunctionalized with l-arginine and RGD peptide through carbodiimide mediated-amide formation to promote bioadhesion of fibroblast and keratinocyte cells as a potential enhancement for wound healing and skin tissue engineering applications.

## Abstract 2

Engineering skin cell bioadhesion through a smart hybrid hydrogel functionalization strategy for wound dressing applications.

## Graphical abstract

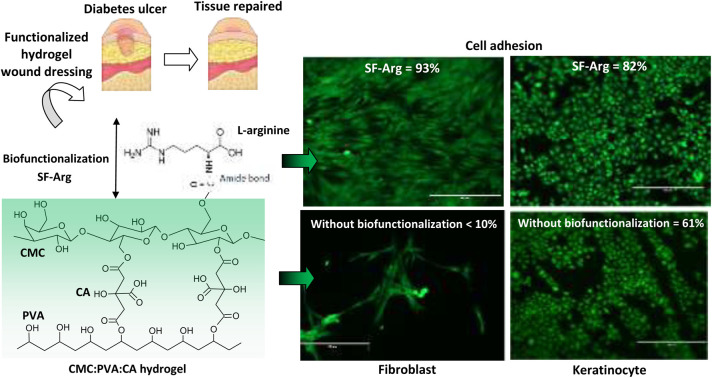

## 1 Introduction

The skin is the body's largest organ and a complex tissue that plays important roles, including a physical protection barrier against external threats, such as harmful chemicals, radiation, and pathogenic microbial agents. Moreover, it performs vital functions in preserving biological homeostasis, assisting in thermoregulation, preventing the extensive loss of body fluids, and as a defense to the body's immune system. Since skin is often in direct contact with the external environment, it permanently suffers from various damages, including burns, contusions, traumas, and diseases, disrupting its vital barrier functions. The interruption of the integrity or malfunction of the skin tissue is regularly referred to as a wound, which must be repaired and restored by the healing process.^1,2^ Skin wound healing depends on a sequence of a well-orchestrated process of multiple phases (hemostasis, inflammation, proliferation, epithelialization, and remodeling). To date, several biomaterials have been developed to improve wound-healing processes, including gauze, fibers, membranes, hydrogels, antimicrobial foams, stimuli-responsive scaffolds, injectable cryogels, bactericidal and antimicrobial films and coatings, and extracellular matrix (ECM) biomimetic scaffolds.^1–3^

In particular, hydrogels have been the preferred choice for wound dressing and promoting chronic wound healing. Hydrogels consist of 3D water-swollen networks of crosslinked hydrophilic polymer chains from natural or synthetic sources.^1–3^ Hydrogels are defined as polymeric materials with three-dimensional (3D) networks made by hydrophilic macromolecular chains crosslinked by chemical groups (–OH, –NH_2_, –COOH) that can absorb a large quantity of water and hold in their interstitial structures to form a swollen stable structure.^3–5^ Crosslinked networks may be established *via* covalent bonding, electrostatic and physical interactions of chemical groups attached to the hydrophilic polymer chain. The types of polymers and crosslinking combinations give rise to limitless possibilities for hydrogels with a broad range of properties and applications. Considering soft tissue engineering and wound healing applications, hydrogels can readily conform to irregularly shaped wounds while retaining moisture and exudate absorptive activity. Moreover, they are versatile and can be engineered through chemical modifications to improve their properties or assign novel functionalities, including multiple biological activities.^4,5^

Generally, naturally-derived polymers (or biopolymers) are more prone to mimic skin tissue's biophysical and biochemical complexity than synthetic artificial polymers.^6,7^ Among naturally-sourced polymers, carboxymethyl cellulose (CMC)-based hydrogels have been selected as skin substitutes and wound dressing due to their intrinsic biocompatibility, biodegradability, and effective adherence to wound sites while exhibiting high exudate absorbency.^3,5–7^ Moreover, CMC promotes wound healing in vivo models, significantly causes the regulation of transdermal water loss, and minimizes the loss of moisture in the wound microenvironment. CMC exhibits prominent water-absorbing ability and enhanced compatibility with skin membranes.^3,6–8^ Although CMC hydrogels can significantly contribute to the chronic wound healing process (*e.g.*, hemostasis, blood clotting, inflammation, cell adhesion, and cellular migration), they do not fulfill all requirements for mimicking skin tissue, such as tunable mechanical properties (*e.g.*, stiffness, flexibility, and elasticity), considering the 4 stages of the healing progression cascade. Unfortunately, such a platform is not available in the literature yet.^3,8^ Thus, using polysaccharide-based hydrogels (*e.g.*, CMC) requires their properties to be improved to assign new functions and features important to assist in wound healing, which can be achieved by combining with other biopolymer or synthetic polymers, and through surface chemical functionalization.^3,9^

In this view, hydrogels and composite materials of CMC and polyvinyl alcohol (PVA) have increased interest over recent years predominantly because of their relative facile synthesis and surface modifications associated with various properties and morphological features. A notable improvement in the mechanical strength, thermochemical stability, and flexibility of the biopolymeric network of CMC has been verified when it is blended and chemically crosslinked with PVA polymer.^10,11^ Therefore, PVA, a broadly available hydrophilic synthetic polymer, has been one of the most frequent choices associated with CMC to produce hydrogel hybrids for biomedical applications. CMC and PVA are miscible and compatible hydrophilic polymers mostly driven by hydrogen bonding.^10^ Moreover, they are non-toxic and environmentally friendly polymers suitable for many advanced biomedical engineering applications. These innovative biopolymer-based blends, composites, and hybrids have been intensively researched because they offer attractive enhanced features in terms of the functionalities and properties mentioned above to mimic skin tissue's characteristics, facilitating the wound healing process and tissue repair.^10–15^

Nonetheless, several hydrogels lack specific binding sites and domains, limiting or hampering direct cell adhesion, which is vital for promoting wound healing.^5,6,9,10^ Hydrogels for wound dressing applications must adhere firmly to cover the open wound and provide a protective microenvironment for wound healing. Thus, they require functionalization to adjust their surface properties to affect the cell adhesion activity and spreading behavior. For those hydrogels, cell-adhesion peptides (CAPs) are required to specifically bind to a cell receptor responsible for the cell adhesion onto the biologically functionalized substrates. They are amino acids (AA) and short peptide sequences (oligopeptides) such as arginine–glycine–aspartic acid (RGD) grafted to the hydrogel to facilitate cell attachment, promoting fibroblast adhesion and improve the wound healing process.^5,6,9,10^ Specifically, RGD is important for cell recognition and attachment sites for several extracellular matrix proteins (ECMs) as well as blood and cell surface proteins. Besides, it has significant regulatory functions in numerous biological activities. RGD peptide sequence is involved in cell attachment, spreading, and focal-adhesion formation with integrin membrane receptors. These overlapped reactions are vital for transmitting signals related to cell behavior and the cell cycle. Hence, RGD-based peptides have been the preferred choice as cell adhesion promoters (“gold standard”) due to their well-known biocompatibility and strong affinity to integrin receptors in cell membranes.^6,16,17^

Despite several publications combining CMC and PVA in the consulted literature, this study reports for the first time the design, synthesis, and characterization of biofunctionalized hydrogels composed of CMC and PVA that were physically crosslinked and chemically modified by citric acid, forming a hybrid polymer network with high water-retention, hydrophilicity, wettability, permeability, cytocompatibility, and hemocompatibility, grafted with adhesion-enhancing biomolecules for improving the wound healing process and skin tissue engineering.

## 2 Material and methods

### 2.1 Materials

Carboxymethylcellulose sodium salt (CMC, degree of substitution, DS = 0.84; average molecular mass, *M*_w_ = 700 000 Da), poly(vinyl alcohol) (PVA, *M*_w_ = 85–124 kDa, degree of hydrolysis 99.3–100%), citric acid (≥99.5%), 2-(*N*-morpholino)ethanesulfonic acid (MES, >99%, low moisture content), 1-ethyl-3-[3-dimethylaminopropyl]carbodiimide hydrochloride (EDC, ≥98%), arginine–glycine–aspartic acid (Arg–Gly–Asp, RGD, ≥97%), l-arginine (≥98%), 3-(4,5-dimethylthiazol-2yl) 2,5-diphenyltetrazolium bromide (MTT, >98%), Triton™ X-100, sodium dodecyl sulfate (SDS, ≥99%), absolute ethanol (≥99.8%), hydrochloric acid (HCl, 37%), sodium chloride (NaCl, ≥ 99%), glutaraldehyde, tannic acid, and osmium tetroxide were purchased from Sigma-Aldrich (USA). Fetal bovine serum (FBS), phosphate-buffered saline (PBS), Dulbecco's Modified Eagle Medium (DMEM), trypsin, soybean trypsin inhibitor, defined keratinocyte serum-free medium (K-SFM), penicillin G sodium, streptomycin sulfate, and amphotericin-b were supplied by Gibco (USA). Calcein-AM was supplied by Invitrogen (USA). Sodium heparin was procured by Cristália (Brazil).

All reagents and precursors were used as received without further purification. Unless specified otherwise, DI-water with a resistivity of 18 MΩ cm (Millipore Simplicity™) was used to prepare the solutions, and the procedures were performed at room temperature (RT, 23 ± 2 °C).

### 2.2. Synthesis and functionalization of hybrid hydrogels

#### 2.2.1 Synthesis of chemically crosslinked hybrid hydrogels

Hybrid hydrogels composed of CMC and PVA were chemically crosslinked by citric acid using a thermal treatment at mild temperatures (referred to as CMC:PVA:CA or WF, without functionalization) as described in the sequence.

Solution of CMC (2% w/v) was prepared by adding the biopolymer powder (4.0 g) to 200 mL of DI-water and stirring at 60 ± 2 °C until complete solubilization was reached. Similarly, the PVA solution was prepared by weighting 4.0 g of polymer and dissolving it in 200 mL of DI-water (2% w/v).

Initially, PVA powder was uniformly dispersed in water at room temperature using an appropriate magnetic stirring to wet out all particles. After 5 min, the solution temperature was increased to 87 ± 2 °C and maintained until the full dissolution of PVA. The polymer solutions were let to cool down to RT before storage and further use.

CMC:PVA mixtures were prepared to obtain the polymer blends by adding 20 mL of PVA solution (2% w/v) to 80 mL of CMC solution (2% w/v) and kept under magnetically stirring. After homogenization for approximately 30 min under moderate stirring, the CA crosslinker agent was added at the concentration of CA/(CMC + PVA) = 25% (w/w) (*m* = 0.5 g) and homogenized for approximately 20 minutes. Next, 10 mL of the solutions were slowly poured into polystyrene Petri dish molds (60 mm diameter), and they were dried at 40 ± 2 °C for 24 h. In the sequence, the samples were submitted to the chemical crosslinking reaction through a thermal treatment at 80 ± 2 °C for 24 h. Then, to remove non-reacted molecules, the hydrogels were placed in DI-water (10 mL: 100 mm^2^) at RT. After 24 h, the hydrogel samples were removed from the water, gently wiped to remove excess liquid, and dried at 40 ± 2 °C until mass stabilization. The hybrids hydrogels were referred to as CMC:PVA:CA.

#### 2.2.2 Functionalization of hybrid hydrogels

For enhancement of bioadhesion, functionalization biomolecules (l-arginine and RGD) were grafted to the hydrogel using EDC as a “zero-length” conjugation agent in MES buffer (pH 5.5 ± 0.1).

The l-arginine solution was prepared by adding amino acid powder (1.26 g) to 12 mL of HCl 0.1 M and adjusting the pH to about 5.5 using HCl 6 M. Then 15 mL of MES buffer (0.2 M, pH 5.5 ± 0.1) was added. The final volume was completed to 30 mL using DI-water.

RGD solution was prepared by adding peptide powder (10 mg) to 2 mL of HCl 0.1 M and adjusting the pH to about 5.5 using NaOH 10 M. Then 4 mL of MES buffer (0.2 M, pH 5.5 ± 0.1) was added. The final volume was completed to 8 mL using DI-water.

EDC solution was prepared by adding 0.474 g of powder to 30 mL of MES buffer (0.1 M, pH 5.5 ± 0.1).

Membranes were functionalized as follows: 10 mL of EDC solution was added to the Petri dish (polystyrene, 90 mm in diameter), and the hydrogel membrane (disc of 60 mm diameter obtained as described in S1) was immersed in EDC solution and incubated at RT for 15 min (1 : 2, COO^−^ : EDC molar ratio) for activation of carboxylic groups of CMC. In the sequence, the EDC solution was removed and replaced by 10 mL of l-arginine or RGD solution (1 : 6, COO^−^ : l-arginine molar ratio and 1 : 0.1, COO^−^ : RGD molar ratio) and kept for 20 h. Then, the functionalized hydrogels were washed with cold PBS (4–6 °C, pH = 7.4 ± 0.2) and immersed for 60 min in the same buffer. Then, samples were washed with distilled water, immersed in distilled water for 24 h, and dried at 40 ± 2 °C for 24 h. The surface functionalized (SF) hydrogels were identified as CMC:PVA:CA_SF-Arg (or SF-Arg, with l-arginine) and CMC:PVA:CA_SF-RGD (or SF-RGD, with RGD).

### 2.3 Characterization of hydrogels

Hydrogels and functionalized hydrogels were extensively characterized based on physicochemical, compositional, and microstructural properties as described in the sequence.

#### 2.3.1 Water absorption and gel fraction tests

For water absorption assessment and gel fraction measurements, the hydrogels were cut into square samples (10 mm × 10 mm), dried at 40 ± 2 °C for mass stabilization, and weighed (initial mass, *W*_0_). Then, the hydrogels (replicates, *n* = 3) were immersed in 10.0 mL of DI-water at RT. After 24 h for reaching the equilibrium, the hydrogels were removed from the solution, softly wiped to remove the excess liquid from the sample, and weighed (swollen mass, *W*_s_). In the sequence, hydrogel samples were dried at 40 ± 2 °C until reaching mass stabilization, and the final weight was recorded (final mass, *W*_f_).

The measurements of weight registered in each step of the process were used to estimate the water absorption (WA) and gel fraction (GF) of the hydrogels using eqn (1) and (2), respectively, as reported in the literature.^3,18^ Each measurement was performed in triplicates (*n* = 3) for both tests, and the results were expressed as the average ± standard deviation.

1 WA (%) = ((*W*_s_ − *W*_0_)/*W*_0_) × 100%

2 GF (%) = ((*W*_0_ − *W*_f_)/*W*_0_) × 100%

#### 2.3.2 Spectroscopic, morphological, surface charge, and thermal analyses

FTIR spectroscopy analysis was recorded with a Nicolet 6700 (Thermo Fischer) spectrometer with background subtraction. The FTIR spectra of hydrogel films were obtained using attenuated total reflectance (ATR) in the wavenumber range of 4000–750 cm^−1^, 32 scans, and 4 cm^−1^ resolution (*n* = 2). XPS analysis of hydrogels was performed using Mg-Kα as the excitation source at 120 W (Amicus spectrometer, Kratos). All peak positions were corrected based on C 1s binding energy (285 eV).

The “point of zero charge” (PZC) of the hydrogels was determined by the “powder addition method” adapted for membranes.^19,20^ Hydrogels of CMC:PVA:CA and CMC:PVA:CA_SF-Arg (8.0 ± 0.5 mg) were immersed in 10 mL of solutions of 0.01 mol L^−1^ of NaCl (background electrolyte) with pH values ranging from 4 to 9 (pH_initial_ adjusted with NaOH and HCl solutions 1.0 and 0.1 mol L^−1^). Then the hydrogels were gently shaken (100 rpm) for 20 h, and the final pH value (pH_final_) of each solution was recorded (pHmeter Quimis, Brazil; *n* = 2). The difference pH_final_ − pH_initial_ (ΔpH) was plotted *versus* pH_initial_ and PZC obtained at the point where ΔpH = 0.

Thermogravimetric and differential scanning calorimetry analyses were performed using the SDT Q-600 instrument (TA Instruments Co.). Hydrogel films of 1.5 ± 0.3 mg were tested at a heating rate of 10 °C min^−1^ from RT to 500 °C. The samples were loaded into an open platinum pan, and an empty cup was used as a reference.

The thermal analyses were performed under the continuous flow of dry nitrogen gas (30 mL min^−1^) (*n* = 2). Scanning electron microscopy images were taken from the surface of hydrogels using FEI-Inspect S50 microscope (FEI Company) coupled with EDX (energy-dispersive X-ray) spectroscopy for elemental image mapping analysis (Genesis, EDAX Inc., USA). Before analysis, samples were coated with a thin gold film by sputtering using a low deposition rate to avoid sample damage.

#### 2.3.3 Thickness, wettability, and permeability tests

The thickness of hydrogels was evaluated using a digital micrometer (Mitutoyo). The results were based on 5 measurements, and the results are expressed as the average ± standard deviation.

The wettability of the hydrogels was evaluated by measuring the contact angle (*ϕ*) formed between the DI-water and the hydrogels. One droplet of DI-water was deposited onto the surface of each hydrogel by a microsyringe at RT, and digital images were acquired immediately after deposition (<5 s). The results represent the average angle between the tangent line at droplets and the surface of the hydrogel membranes (*n* = 3).

The permeability to water vapor was determined by measuring the water vapor transmission using an adaptation of the ASTM E96/E96M-16 (Standard Test Method for Water Vapor Transmission of Materials). The hydrogel membrane was firmly attached to the top of a plastic dish containing 2.0 mL of distilled water and the weighted (*W*_i_). The vials were then placed under controlled room conditions (temperature = 19 ± 2 °C, relative humidity of 50 ± 5%), and the dish assembly was weighed after 24 h (*W*_24h_). The water vapor transmission after 24 h (WVT) was determined according to eqn (3). The tests were performed in duplicate, and the results are expressed as the average.

3 WVT = (*W*_24h_ − *W*_i_)/(*A* × *T*)

where: *A* is the cup mouth area (7.5 cm^2^), and *T* is the time exposed to controlled climatic conditions (24 h, 1 day).

### 2.4 Biological tests

All biological tests were conducted according to ISO 10993-5:2009/(R)2014 (Biological evaluation of medical devices: tests for *in vitro* cytotoxicity). Before the experiments, the samples were sterilized by UV radiation for 40–60 min.

Cytotoxicity of hydrogels based on MTT (3-(4,5-dimethylthiazol-2-yl)-2,5-diphenyltetrazolium bromide) protocols and hemolysis index tests (Ethical committee approved project protocol no. 22/2018) were performed to assess the *in vitro* compatibility of hydrogels. Bio-adhesion of skin cells (fibroblasts and keratinocytes) was evaluated by seeding and incubating cell cultures on hydrogels.

Cell viability and attachment were measured using fluorescent images obtained using fluorescence microscopy after cell staining with calcein-AM. Also, SEM images of the surface of the hydrogel after incubation with cells were obtained. The details of each protocol are presented in the following sections.

#### 2.4.1 MTT cytotoxicity tests

Human embryonic kidney cells (HEK293T, American Type Culture Collection – ATCC^®^ CRL-1573™) were provided by the Federal University of Minas Gerais (UFMG, Profa. Maria de Fátima Leite). Human malignant melanoma cells (A375, ATCC^®^ CRL-1619™) were purchased from Brazilian Cell Repository (Banco de Células do Rio de Janeiro: BCRJ, Brazil). Fibroblasts isolated from the lung tissue (MRC5) were provided by the Fundação Ezequiel Dias (FUNED, Profa. Luciana M. Silva). The cytotoxicity of the hydrogels was evaluated using the direct contact 3-(4,5-di-methyl-2-thiazolyl)-2,5-diphenyltetrazolium bromide method as previously described by our group.^21^ Cells were cultured and tested in a Dulbecco's Modified Eagle Medium (DMEM, pH 7.4 ± 0.2), and cells were exposed to 24 h in contact with samples. Percentage cell viability was calculated according to eqn (4). The values of the controls (wells with cells, and medium without samples) were set to 100% cell viability.

4 Cell viability (%) = (absorbance of sample and cells/absorbance of control) × 100%

#### 2.4.2 Cell seeding and cultivation – fibroblasts

Human dermal fibroblasts (HDFa, Gibco – C0135C) were provided by the Federal University of Minas Gerais (UFMG, Prof. Dawidson Gomes). HDFa cells were cultured in DMEM at 37 °C in the atmosphere of 95% air and 5% CO_2_.

Hydrogels (WF, SR-Arg, and SF-RGD) were swollen in saline solution (PBS 1×) for 10 min and were cut into 11 mm diameter samples. Before cell seeding, the prepared circular membranes of hydrogels were placed in the wells of 24-well plates with 1 mL of DMEM supplement with 10% FBS and 1% antibiotic–antimycotic and incubated for 24 h at 37 °C with a controlled atmosphere of 5% CO_2_ and 95% relative humidity.

HDFa cells were detached using trypsin and counted before seeding on hydrogels. To perform cell adhesion analysis, 1 × 10^5^ cells per well were seeded and incubated in a humidified atmosphere of 95% relative humidity and 5% CO_2_ at 37 °C for 6 days (*n* = 3). The culture medium was changed every two days.

#### 2.4.3 Cell seeding and cultivation – keratinocytes

Primary epidermal keratinocytes, normal, human, adult (HEKa, ATCC^®^ – PCS-200-011™)) were provided by the Fundação Ezequiel Dias/Centro Federal de Educação Tecnológica de Minas Gerais (FUNED/CEFET, Prof. Sidney Nicodemos). HEKa were cultured in a defined keratinocyte serum-free medium (K-SFM) at 37 °C in the atmosphere of 95% air and 5% CO_2_.

Hydrogels (WF, SR-Arg, and SF-RGD) were swollen in saline solution (PBS 1×) for 10 min and cut into 11 mm diameter samples. Before cell seeding, the prepared circular membranes of hydrogels were placed in the wells of 24-well plates and incubated with 1 mL of K-SFM with growth supplement and 1% antibiotic–antimycotic and incubated for 24 h at 37 °C with a controlled atmosphere of 5% CO_2_ and 95% relative humidity.

HEKa cells were detached using trypsin and counted before seeding on hydrogels. A soybean trypsin inhibitor was used (250 mg L^−1^). For the analysis of cell adhesion, 2 × 10^5^ cells per well were seeded and incubated in a humidified atmosphere of 95% relative humidity and 5% CO_2_ at 37 °C for 7 days (*n* = 3). The culture medium was changed every two days.

#### 2.4.4 Cell staining

To assess the cell viability and adhesion, live staining was performed with calcein acetoxymethyl ester (2.5 μM of calcein-AM) after 6 (HDFa) or 7 (HEKa) days of cultivation. The images of calcein-stained cells were captured by a fluorescence microscope using EVOS imaging systems (Thermo Fisher Scientific). Positive control was cells cultivated in wells without hydrogel membrane (polystyrene; surface: standard, for adherent cells; flat base; sterile; Sarstedt, Germany), and fluorescent images of three different samples (total of 12 images) were analyzed using Graph Pad Prism 9 software to determine the cell adhesion (percentage of area occupied by cells). Data were presented as average ± standard deviation. Statistical analyses were accomplished by one-way analysis of variance (ANOVA), and *p*-values of 0.05 were considered statistically significant.

#### 2.4.5 Scanning electron microscopy (SEM) analysis

The morphology of HDFa on hydrogel films after 6 days of cultivation was analyzed by SEM (FEI–Inspect S50 microscope, FEI Company). Briefly, HDFa-seeded films were fixed with glutaraldehyde (2.5%) for 2 h. On the next day, a second fixative was performed using tannic acid and osmium tetroxide. After dehydration with absolute ethanol, samples were critical-point dried (Center of Microscopy – UFMG) and coated with gold sputter before SEM examination.

#### 2.4.6 *In vitro* hemolysis ratio measurement

The hemolytic index (HI) of the samples was determined according to the methodology proposed by Ghorpade *et al.*^22^ with modifications. Hydrogels with 1.0 cm × 1.0 cm were allowed to swell in the phosphate buffer saline for 1 h at 37 °C (*n* = 3). After that, PBS was removed, and 250 μL mice blood (isogenic male BALB/c adult mice, Ethical committee approved project protocol no. 22/2018) with heparin (4 UI mL^−1^) was added. The systems were left undisturbed for 20 min, and then, the hemolysis process was stopped by adding 1.75 mL of 0.9% NaCl saline, followed by incubation of the samples for 1 h at 37 °C. Next, the mixtures were centrifuged at 4000 rpm for 5 min, and the absorbance of the clear supernatant was measured at 545 nm using a spectrophotometer (Spectramax M, Molecular Devices). HI values were calculated according to eqn (5).

5 HI (%) = [(*A*_sample_ − *A*_(−)control_)/(*A*_(+)control_ − *A*_(−)control_)] × 100%

where: *A* is the absorbance, (+) control = mixture of de 250 μL of blood and 1.75 mL double-distilled water; and (−) control = mixture of de 250 μL of blood and 1.75 mL of 0.9% NaCl saline.

## 3 Results and discussion

### 3.1 Hydrogel characterization

**Fig. 1:**
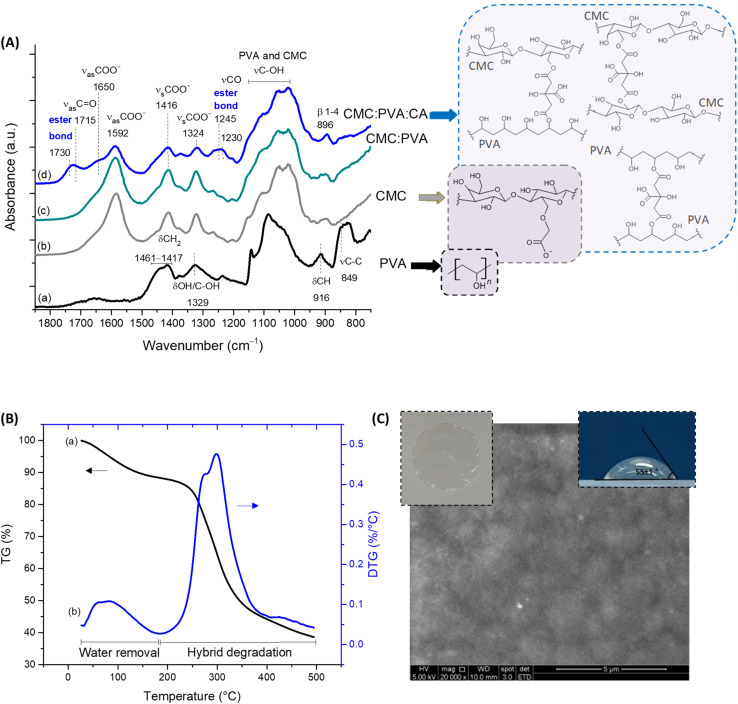
(A) FTIR spectra (range 1850–750 cm^−1^) obtained for (a) PVA, (b) CMC, (c) CMC:PVA (physical crosslinking), and (d) CMC:PVA:CA (chemical crosslinking), including chemical formulas of pure polymers and the schematic representation of CA-mediated crosslinking reactions based on esterification for forming hydrogels (CMC–PVA, CMC–CMC, and PVA–PVA). (B) (a) TG and (b) DTG analysis of CMC:PVA:CA hydrogel. (C) Digital image (insert, top left), contact angle (insert, top right), and SEM image (200 00×, scale bar = 5 μm) of CMC:PVA:CA hydrogel.

In a macroscopic visual analysis, CMC:PVA:CA hybrid hydrogel was optically transparent and uniform, without evidence of phase separation or segregation (Fig. 1C, insert top left), with a thickness (*t*) of 53 ± 6 μm. At the microscopic level (Fig. 1C, SEM image), it can be observed that the hydrogels were relatively uniform, with no segregation or failure of miscibility with homogenous surface morphology. FTIR spectra (Fig. 1A) of the hybrid of CMC and PVA crosslinked with CA were mostly dominated by the bands of carboxymethylcellulose, the main component of the hydrogel (CMC : PVA 80 : 20). Upon the chemical crosslinking reaction, it was observed in the regions at 1730–1725 cm^−1^ and 1250–1240 cm^−1^ the vibrations related to ester bonds (R_1_–COO–R_2_) formation, demonstrating the esterification reaction between carboxylic groups of the crosslinker (CA) with hydroxyl groups (OH) of CMC and PVA polymers.^3,23^ Crosslinking reactions mediated by citric acid involving CMC–PVA, CMC–CMC, and PVA–PVA polymer chains were thermodynamically and statistically expected in blends considering that both polymers possess hydroxyl groups (–OH) and separately (*i.e.*, pure hydrogels, not blended), they also demonstrated that the esterification reaction with CA has occurred (ESI, Fig. S1 and S2†). The non-crosslinked samples were water-soluble as they relied on physical interactions between the polymers. Conversely, the hybrid with CA presented a water absorption of 78 ± 7% and gel fraction of 97 ± 3%, consistent with the formation of ester bonds rendering water-stability for the crosslinked hybrid polymeric network.

Regarding thermal stability profiles (Fig. 1B), for CMC:PVA:CA hybrids, two main stages of mass loss were observed up to 500 °C. The first is related to water loss from the beginning of heating up to 200 °C involving the removal of moisture (up to 110 °C) and vaporization of bound water tightly attached to the polymer matrix (110–200 °C). The mass loss involved was about 10%, characteristic of the hydrophilic network. The second region of significant mass loss was detected between 200 and 500 °C associated with the thermal events degradation of polymers. These findings evidenced that these hydrogels presented chemical and thermal stabilities suitable for wound dressings.^3,23^

Furthermore, bearing in mind the wound dressing and skin tissue applications of the hydrogels, the properties of permeability and surface wettability/hydrophilicity are important to maintain a balanced moisture microenvironment at the wound site. These characteristics play a pivotal role in the cell response to the biomaterial. The permeability evaluated by water vapor transmission WVT = 338 ± 5 g m^−2^ d^−1^ is compatible with the range for normal (healthy) skin tissue (∼200 g m^−2^ d^−1^) and commercial wound dressings (76–9360 g m^−2^ d^−1^).^23,24^ To assess the wettability and hydrophilicity of the hybrids, the contact angle was used (55 ± 1°, Fig. 1C, insert, top right), indicating a moderate hydrophilic material that is consistent with the optimal values reported for enhancing cell attachment, adhesion, and proliferation (55°–85°).^14,25^ It should be highlighted that the numerous interactions occurring at the cell–biomaterial interfaces are very complex, where hydrophilic and hydrophobic forces are present and, therefore, an in-depth evaluation is beyond the scope of this study. To this end, these findings demonstrated that, from a physicochemical strategy, these hydrogels could be potentially suitable for wound dressing and skin repair substitutes.

### 3.2 Hydrogel functionalization

**Fig. 2:**
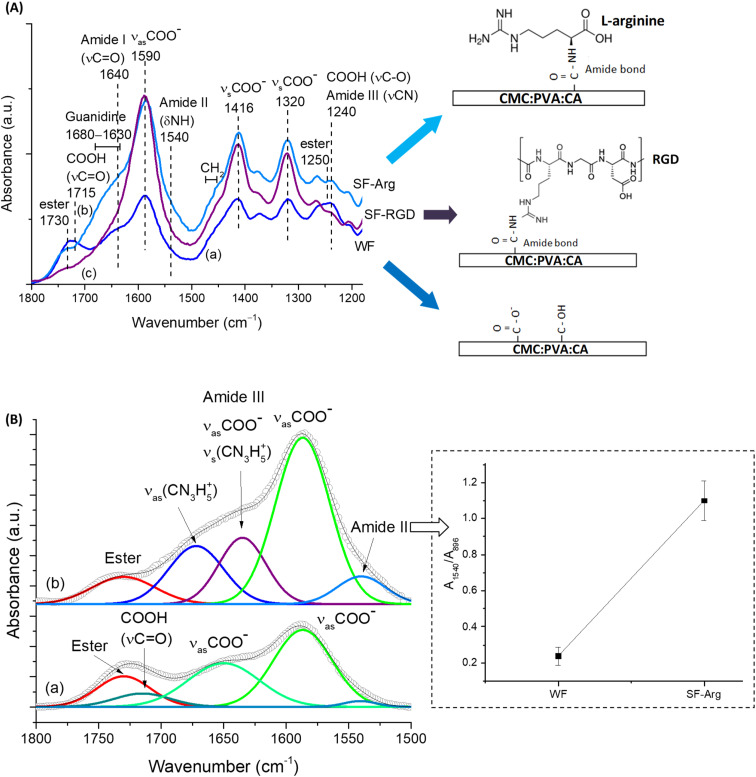
(A) FTIR spectra of (a) CMC:PVA:CA hydrogel (WF), (b) SF-Arg, and (c) SF-RGD with the schematic representation of functional groups of pristine hydrogel and membranes functionalized with grafted biomolecules (l-arginine and RGD). (B) Deconvolution of WF and SF-Arg peaks in the range of 1800–1500 cm^−1^ demonstranting the formation of amide bonds. Inset: *A*_1540_/*A*_896_ ratio.

To promote and enhance cell attachment to CMC:PVA:CA hybrid hydrogels for diabetic wound healing, the amine-based molecule was selected since –NH_2_ groups have been reported to promote cell adhesion and proliferation.^26,27^ In this sense, l-arginine amino acid was used as a hydrogel modifier because, besides favoring the bioadhesion, it is also expected *in vivo* to contribute to the production of nitric oxide (NO), which can support vascularization, to the promotion of insulin secretion, and to exhibit good anti-thrombogenic activity.^28,29^ The tripeptide “arginine–glycine–aspartic acid” (RGD) was used as a reference as it is the most reported active peptide sequence affecting cell attachment and non-cytotoxic. The schematic representations of the biofunctionalized hydrogels are displayed in Fig. 2A, focusing on the amidation reaction (R_1_–NH_2_ + R_2_–COOH → amides).

The functionalization of CMC:PVA:CA membrane with l-arginine and RGD was primarily monitored using FTIR spectroscopy within the range from 1800 to 1100 cm^−1^ (Fig. 2A) that contains the most relevant peaks related to the EDC-mediated reaction. Peaks associated with carboxylate groups (R–COO^−^) of CMC at 1590, 1416, and 1320 cm^−1^ were identified in all spectra.

Also, for unmodified membrane (Fig. 2A(a)), peaks of ester bonds (1730 and 1250 cm^−1^) overlapped with carboxylic groups from protonated CMC (1715 and 1240 cm^−1^) were also observed. On the other hand, for modified membranes, carboxylic peaks (R–COOH) were not perceptible as the functionalization procedure increased pH above p*K*_a_ of CMC (pH > p*K*_a_, R–COOH → R–COO^−^), remaining observable mostly the peaks of the esterification reaction between citric acid and polymers. After the EDC-mediated crosslinking reaction of carboxylic groups of CMC with amino groups of l-arginine (Fig. 2A(b)) forming amide bonds (N–C=O), it was noted the appearance of the vibrations of amide I (νC = O), amide II (δNH and νCN), and amide III (νCN and δNH) at 1640, 1540, and 1240 cm^−1^, respectively. Moreover, the presence of l-arginine in CMC:PVA:CA_SF-Arg hydrogel membrane was identified by detecting guanidine peaks overlapped with amide I vibration and the bands at 1475–1455 cm^−1^ associated with the –CH_2_ groups of the amino acid aliphatic chain.^30^ These findings evidenced the effective biofunctionalization of hydrogels with l-arginine (CMC:PVA:CA_SF-Arg). For FTIR analysis of CMC:PVA:CA_SF-RGD (Fig. 2A(c)), due to the relatively much lower concentration of RGD peptide (60-fold lower than l-arginine), the functionalization of the hydrogel membrane with RGD could not be accurately noticed by FTIR. Nonetheless, RGD modification of carboxylic polymers, including CMC, has been extensively reported for grafting this adhesion-promoter peptide to hydrogels and composites in biomedical applications through EDC-mediated reaction forming amide bonds.^10,17,31^

To present a more in-depth and accurate analysis using FTIR of the biofunctionalization of CMC:PVA:CA hydrogel with l-arginine (CMC:PVA:CA_SF-Arg), both spectra were deconvoluted (Fig. 2B). In SF-Arg membrane, the band of amide II at 1540 cm^−1^ could be clearly observed. The amide I, at about 1640 cm^−1^, is overlapped with the bands of symmetric stretching of guanidine (ν_s_CN_3_H_5_^+^) at 1635 cm^−1^ (ref. 32) and asymmetric stretching of ν_s_COO^−^ at 1650 cm^−1^. Moreover, the amide II vibration was quantified based on the ratio of the absorbance of this band (*A*_1540_) and the reference band of CMC (β1-4 glycoside bond at 896 cm^−1^, *A*_896_) (inset in Fig. 2B). The increase of the ratio of amide II band also demonstrated the occurrence of the EDC-mediated crosslinking reaction through amidation for biofunctionalization.

**Fig. 3:**
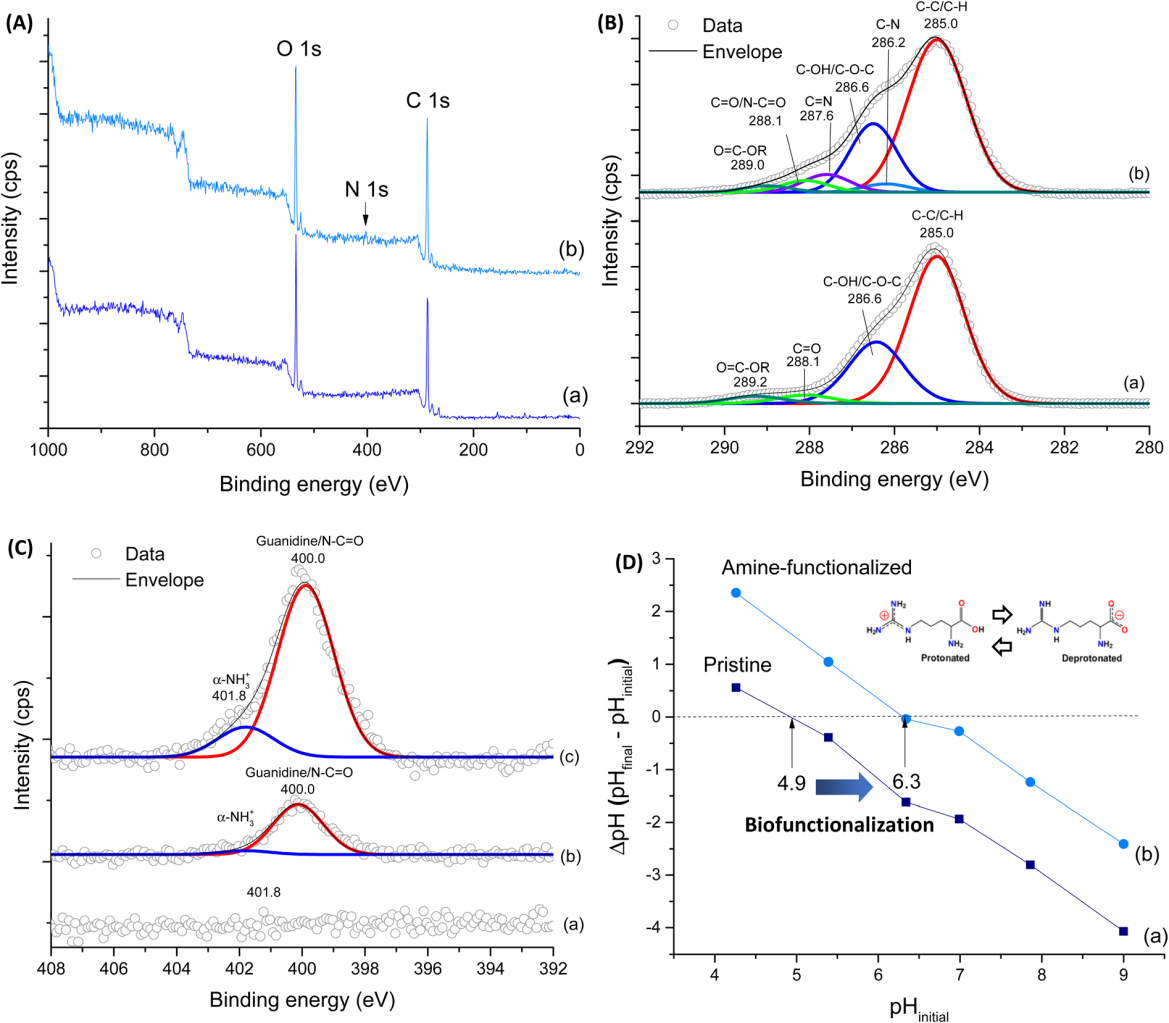
(A) XPS survey spectra of (a) WF and (b) SF-Arg. (B) high-resolution XPS spectra of C 1s for (a) WF and (b) SF-Arg. (C) high-resolution XPS spectra of N 1s region for (a) WF, (b) SF-RGD, and (c) SF-Arg. (D) PZC studies for (a) WF and (b) SF-Arg.

To further corroborate the effective functionalization of CMC:PVA:CA membranes (also referred to as WF, without functionalization), XPS survey scan spectra of the pristine and modified sample (SF-Arg) were performed (Fig. 3A). The spectrum of CMC:PVA:CA showed two main peaks corresponding to C 1s (∼285 eV) and O 1s (∼533 eV). In addition to peaks C 1s and O 1s, CMC:PVA:CA_SF-Arg showed one another peak corresponding to N 1s (∼400 eV). The surface chemical composition of modified and unmodified membranes was calculated (Vision Processing Software, Kratos) and is listed in Table 1. The results showed that in SF-Arg and SF-RGD the atomic nitrogen concentration was 3.5 and 1.3 at%, respectively, demonstrating the incorporation of nitrogen species in biofunctionalized materials, as they are present in l-arginine and RGD modifiers and absent in CMC:PVA:CA hydrogels. For a more detailed analysis, the high-resolution XPS spectra of C 1s and N 1s regions were performed to investigate the functional groups in the hydrogels. Fig. 3B shows the C 1s high-resolution spectra for CMC:PVA:CA and CMC:PVA:CA_SF-Arg. For the CMC:PVA:CA membranes (Fig. 3B(a)), C 1s peaks occurred at 285.0 eV (C–H and C–C bonds), 286.6 eV (C–OH and C–O–C bonds), 288.1 eV (C=O bonds), and 289.2 eV (O=C–OR, bonds from acid and esters).^23^ Upon functionalization (Fig. 3B(b)), new peaks were observed at 286.2 eV and 287.6 eV associated with C–N and C=N, evidencing the modification by the l-arginine amino acid. The peaks of amide bonds (N–C=O, from EDC-crosslinking reaction) and COOH/COO^−^ groups from the amino acid carboxyl group were overlapped with the carbonyl peaks of CMC at 288.1 eV and O=C–OR at 289.0 eV, respectively. As expected, no signal was detected for the non-functionalized CMC:PVA:CA hydrogel when analyzing the N 1s region (Fig. 3C(a)). On the contrary, the N 1s spectrum of the membrane modified with l-arginine (Fig. 3C(c)) and RGD (Fig. 3C(b)) exhibited two peak components at 400.0 eV and 401.8 eV. The first one is representative of the three nitrogen atoms of the guanidine group and amide linkage (N–C=O), while that at 401.8 eV arises from the protonated amine (α-NH_3_^+^).^33,34^ These results validated the effective incorporation of l-arginine and RGD peptide functionalization agents in the pristine CMC:PVA:CA hydrogels, which will be responsible for promoting bioadhesion with skin cells.^31^

**Table 1:** Surface chemical composition of hydrogels

Hydrogel sample	XPS (elemental atomic concentration)		
	C 1s	O 1s	N 1s
WF	75.8 ± 2.5%	24.3 ± 2.5%	Not detected
SF-Arg	69.9 ± 0.1%	26.7 ± 0.3%	3.4 ± 0.4%
SF-RGD	68.2 ± 2.0%	30.6 ± 0.3%	1.3 ± 0.1%

For further support biofunctionalization analysis and its effects on the surface charge of hydrogels, PZC measurements of hydrogels were performed (Fig. 3D). The PZC values were 4.9 and 6.3 for WF and SF-Arg, respectively, as indicated by the points of intersection at the line of ΔpH = 0. These results agree with the prevalence of COOH ionizable groups (R–COO^−^/H^+^) of the unmodified hydrogel.^35^ It also indicated the effect of grafting l-arginine amino acid, as adding amine-based functional groups to the hydrogel surface shifted PZC to higher values, which has affected the bioadhesion properties of membranes.^36,37^

**Fig. 4:**
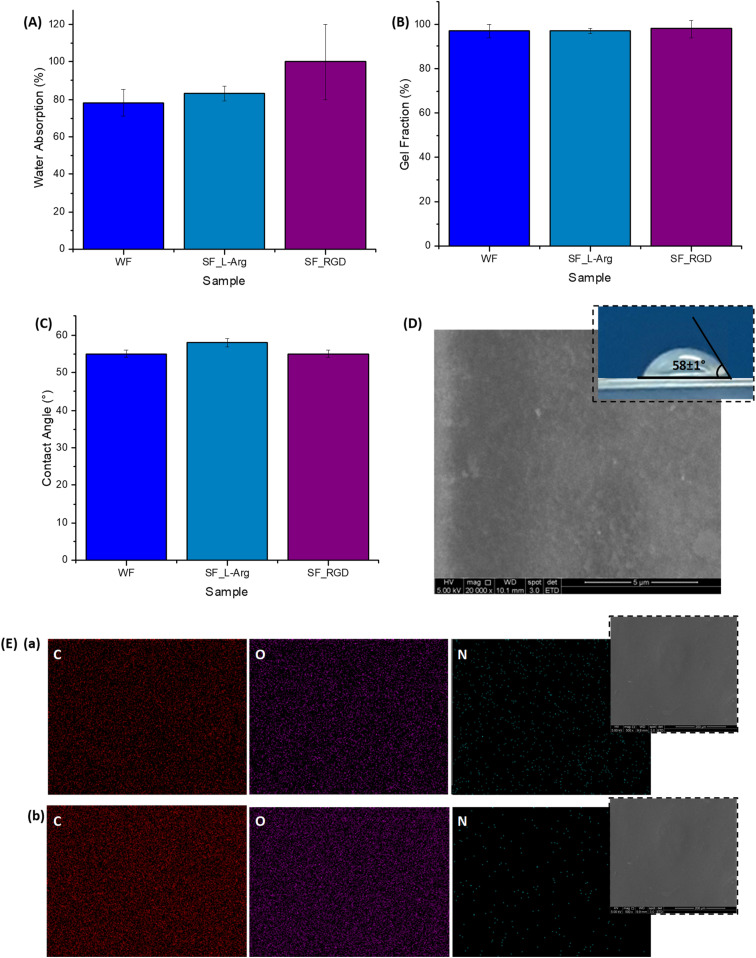
(A) Water absorption, (B) gel fraction, and (C) contact angle values of reference (WF) and functionalized hydrogels (SF-Arg and SF-RGD). (D) SEM image of SF-Arg surface (200 00×, scale bar = 5 μm; insert contact angle). (E) EDX image mapping of C, O, and N obtained at (a) SF-Arg and (b) WF surfaces (insert SEM image of the region of analysis; 5000×, scale bar = 20 μm).

Importantly, water absorption, gel fraction, and contact angle (Fig. 4A–C, inset Fig. 4D) characteristics of the hydrogel membranes were not significantly changed after functionalization with l-arginine and RGD. These findings were anticipated as the selection of the modifiers was designed to preserve these important physicochemical properties of hydrogels (liquid uptake/retention, water-stability, and surface wettability) for wound dressing applications. Regarding the contact angle measurements, as previously described, the similar values found are crucial for cell adhesion, considering that very low or high hydrophilic surfaces do not favor cell attachment/spreading due to the complex balance of interactions at biointerfaces.^14,25,31^

Regarding morphological features, the SEM image of the SF-Arg modified hydrogel (Fig. 4D) exhibited a refined surface morphology compared to CMC:PVA:CA. In addition, a comparison of EDX elemental image mapping directly over the surface of the hydrogel surface before (Fig. 4E(b)) and after the functionalization with l-arginine (Fig. 4E(a)) indicated the presence of C, O, and N in the modified sample, showing that N was uniformly distributed ascribed to the homogeneity of the grafting of the biomolecule to the hydrogel. Thus, the consolidation of these findings confirmed the effective biofunctionalization by l-arginine and RGD of the CMC:PVA:CA hybrid hydrogels to be tested as bioadhesion promoters in the subsequent biological assays.

### 3.3 Cytotoxicity, hemolysis, and cell adhesion

**Fig. 5:**
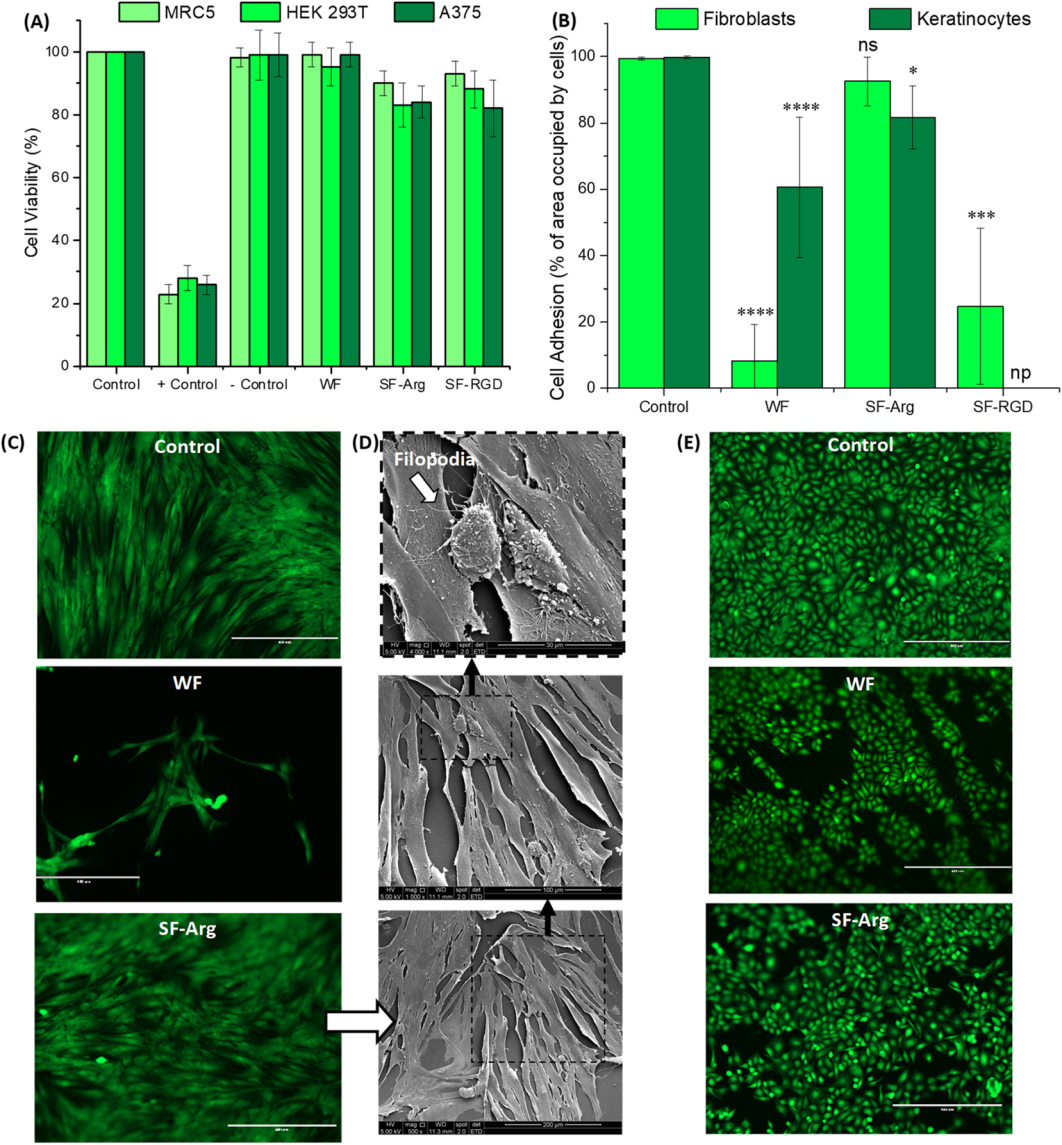
(A) Cell viability results of cell cultures based on MTT protocol after 24 h of incubation with hydrogels. (B) Percentage of the area occupied by cells on hydrogels after 7 days of cultivation (np = not performed; * significant difference compared with the control group; ns = not significant, **** = *p* < 0.0001, *** = *p* < 0.001, and * = *p* < 0.05; ANOVA one-way test). (C) Calcein-AM-stained fluorescence images of (C) fibroblasts and (E) keratinocytes on WF and SF-Arg hydrogels in comparison to control (PS plate) (scale bar = 400 μm). (D) Morphological assessment of fibroblasts on SF-Arg hydrogels with SEM. (from top to bottom: 4000×, scale bar = 30 μm; 1000×, scale bar = 100 μm; and 500×, scale bar = 200 μm).

The cell viability was evaluated by quantitative MTT assay. All the hydrogels exhibited favorable cytocompatibility with cell viability >80% after being cultured for 24 h (Fig. 5A). The results confirmed that the biofunctionalization was non-toxic to all cell lines tested, HEK 293T, A375, and fibroblast cells (MCR5).

Cell adhesion to the surface of the hydrogel was quantitatively evaluated based on the percentage of area occupied by cells (Fig. 5B, C and E) based on fluorescent imaging of live cells stained with calcein-AM, which also demonstrated that modified hydrogels were not cytotoxic. For fibroblasts (HDFa, the biofunctionalization was effective for improving bioadhesion with values not significantly different from control (PS plate, 99%) and about 11-fold higher than CMC:PVA:CA hydrogel (WF = 8.2% and SF-Arg = 92.6%). The potential of RGD as an active peptide for cell attachment was also observed, and it promoted cell adhesion only 4× smaller than SF-Arg with a molar concentration 60× lower. These results of enhanced bioadhesion were assigned to the effective chemical biofunctionalization of the hybrids, considering that wettability and other properties, which influence cell attachment and spreading, were not affected by grafting these biomolecules to the hydrogel polymeric network.

Qualitative analysis based on SEM images (Fig. 5D), showed that the adhered fibroblasts on SF-Arg hydrogels presented a flattened aspect and displayed their typical elongated and spindle-like morphology while evidencing their preference for aligning in parallel to one another. In addition, the presence of growing filopodia (white arrow in Fig. 5D) represents a sign of cell activity for adhesion, migration, formation of cell–cell contacts, and wound healing.^27^ Therefore, due to the excellent cell attachment activity promoted by l-arginine and its viability and cost-effectiveness for potentially reaching clinical applications, the other experiments were only conducted with SF-Arg hydrogel.

For keratinocytes, another skin cell type, the attachment to “bare” hydrogel was enhanced compared to fibroblasts (61% *versus* 8%). Also, the chemically modified hydrogels with l-arginine increased the bioadhesion (>80%). However, the differences observed in the attachment behavior of these two important skin cells on unmodified and functionalized hydrogels might be explained based on the cell adhesion molecules (CAM). CAMs are a group of cell surface proteins, including integrins and cadherins, that are involved in the binding of cells to the extracellular matrix and with other cells, which is cell-type dependent, resulting in different expression profiles for fibroblasts and keratinocytes.^38^

Based on the promising results of *in vitro* biological assays (*i.e.*, MTT and bioadhesion) to evaluate the hydrogels, the hemocompatibility responses of the pristine hydrogel (*i.e.*, CMC:PVA:CA, unmodified) and l-arginine functionalized sample (CMC:PVA:CA_SF-Arg) were assessed through hemolysis tests. Besides, from a translational perspective to clinical applications in the future of these hydrogels as wound dressings, RGD-modified materials would be much more expensive than the analogs with widely commercially available l-arginine amino acid. According to the literature,^28^ the hemolysis index (HI) represents the extent of red blood cells broken by the sample in contact with whole blood. The smaller the HI value, the better the blood compatibility of the biomaterial, where HI values should be below 5.0%. Hence, the HI values for CMC:PVA:CA and CMC:PVA:CA_SF-Arg were 1.2% and 1.0%, respectively, similar to those obtained for the reference standard biomaterial collagen (HI = 1.1%). In this sense, the hydrogels showed hemocompatibility and could be considered nonhemolytic.

## 4 Conclusions

This study demonstrated that hybrid hydrogel membranes composed of CMC (80), PVA (20), and chemically crosslinked with citric acid (CA, 25%) were effectively produced and extensively characterized. The results (FTIR, XPS, SEM/EDX, and PZC) confirmed the biofunctionalization of CMC:PVA:CA hydrogels with l-arginine and RGD based on the covalent conjugation of biomolecules using the EDC-chemistry producing cytocompatible hydrogel biomaterials. Moreover, the biofunctionalization of hybrids enhanced bioadhesion due to the several contributions of chemical and biochemical interactions between the hydrogels and cells at the biointerfaces, considering that the contact angle and other properties were not significantly altered compared to “bulk” analogs (*i.e.*, without functionalization). Besides the high cell viability responses (*i.e.*, cytocompatibility > 80%) and the best bioadhesion results, l-arginine functionalized hybrid hydrogels also presented a nonhemolytic behavior highly required for wound healing applications. In summary, considering the translational medicine strategy, these findings demonstrated that CMC:PVA:CA_SF-Arg hydrogel is a promising, viable, and cost-effective material for chronic wound treatment applications and skin tissue engineering.

## Author contributions

Alexandra A. P. Mansur: methodology, investigation, formal analysis, writing – original draft. Michele A. Rodrigues: methodology, investigation, formal analysis, writing – original draft. Nádia S. V. Capanema: investigation. Sandhra M. Carvalho: methodology, investigation. Dawidson A. Gomes: conceptualization, supervision, writing – original draft. Herman S. Mansur: conceptualization, writing – original draft, supervision, funding acquisition, project administration.

## Conflicts of interest

The authors have no competing interests to declare relevant to this article's content.

## Supplementary Material

RA-013-D3RA01519J-s001
