# Supplementary material for: Functionalized bioadhesion-enhanced carboxymethyl cellulose/polyvinyl alcohol hybrid hydrogels for chronic wound dressing applications
Source: RSC Adv. 2023 Apr 28;13(19):13156–68. doi: 10.1039/d3ra01519j (PMC10140670; doi:10.1039/d3ra01519j)
Supplement: RA-013-D3RA01519J-s001 [file RA-013-D3RA01519J-s001.pdf]

Electronic Supplementary Material (ESI) for RSC Advances.
This journal is © The Royal Society of Chemistry 2023

Electronic Supplementary Information

Functionalized Bioadhesion-Enhanced Carboxymethyl Cellulose/Polyvinyl Alcohol Hybrid Hydrogels for Chronic Wound Dressing Applications

Mansur, A. A. P. [a#], Rodrigues, M. A.[b#], Capanema, N. S. V. [a], Carvalho, S. M. [a], Gomes, D. A.[b], Mansur, H. S.[a*]

[a]*Center of Nanoscience, Nanotechnology and Innovation - CeNano^{2}I, Department of Metallurgical and Materials Engineering, Federal University of Minas Gerais, Brazil*

[b]*Department of Biochemistry and Immunology, Federal University of Minas Gerais, Brazil*

*e-mail: *hmansur@demet.ufmg.br*

[#]*These authors contributed equally to this work*

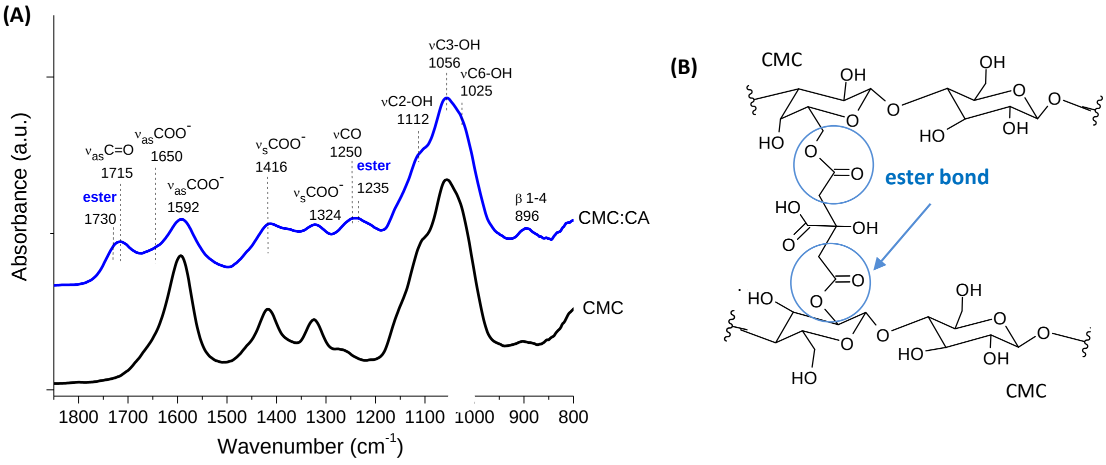

Figure S1 – (A) FTIR spectra of CMC and CMC crosslinked with citric acid (CMC:CA). (B) Schematic representation of CMC:CA crosslinking with the formation of di-ester bonds.

* To whom correspondence should be addressed: Federal University of Minas Gerais, Av. Antônio Carlos, 6627 – Escola de Engenharia, Bloco 2 – Sala 2233, 31.270-901, Belo Horizonte/MG, Brazil; Tel: +55-31-34091843; E-mail: hmansur@demet.ufmg.br (H. Mansur)

FTIR spectrum of CMC (Figure S1A, CMC) shows the characteristic bands of CMC in the range of 1850-800 cm^{-1} and their respective assignment. The region between 1800-1200 cm^{-1} contains the contributions from carboxylate groups of CMC ($\nu_{as}COO^-$ at 1650 and 1592 cm^{-1} and ν_sCOO^- at 1416 and 1324 cm^{-1}). The C–O vibrations from hydroxyl groups (C2-OH at 1112 cm^{-1}, C3-OH at 1056 cm^{-1}, and C6-OH at 1025 cm^{-1}) were also detected and the vibration of glycoside bonds (β 1-4) was observed at 896 cm^{-1}.

After crosslinking with CA (Figure S1A, CMC:CA), carboxylic acid bands from CMC (–COOH bands at 1715 cm^{-1} and 1250 cm^{-1}) were also observed associated with the protonation-deprotonation of carboxylic groups (R-COOH/R-COO^-) in the polymer chains due to the acidification promoted by citric acid. The ester bands (R_1-COO-R_1, R_1 = CMC) formed in the chemical crosslinking reaction CMC:CA were observed at 1730 cm^{-1} and 1235 cm^{-1}, overlapping the bands related to the analogous vibration of COOH in CMC. The schematic representation of the formation of ester bonds between CMC chains mediated by citric acid is depicted in Figure S1B.

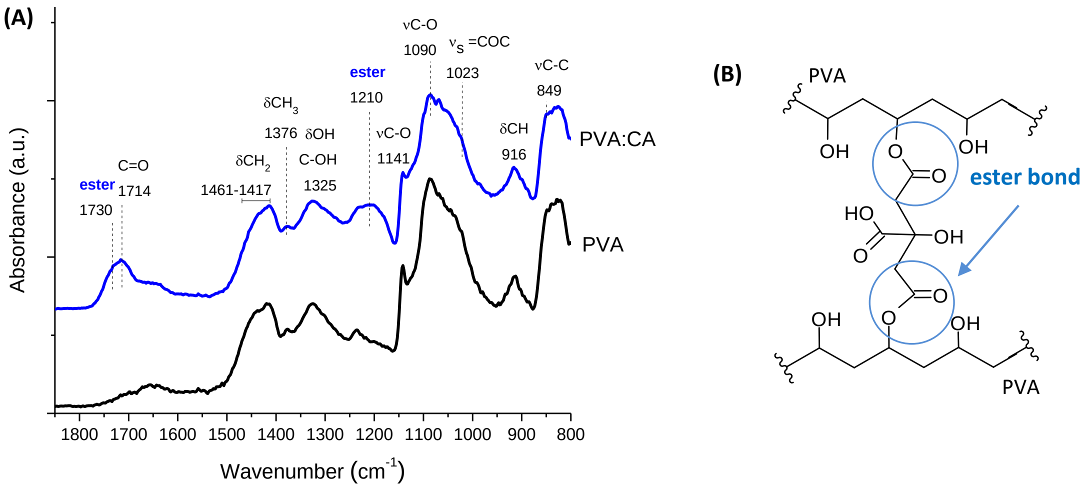

Figure S2 – (A) FTIR spectra of PVA and PVA crosslinked with citric acid (PVA:CA). (B) Schematic representation of PVA:CA crosslinking with the formation of di-ester bonds.

FTIR spectrum of PVA (Figure S2A, PVA) shows the characteristic bands of PVA in the range of 1850-800 cm^{-1} and their respective assignment. The vibrations of alkyl groups (δCH_2) were observed at 1461-1417 cm^{-1} and 916 cm^{-1}. The vibrations of hydroxyls (C-OH) were detected at 1329 cm^{-1} (δOH), and 1141 cm^{-1} and 1090 cm^{-1} (νC-O). The bands at 1714 cm^{-1}, 1376 cm^{-1}, and 1023 cm^{-1} are associated with νC=O, δCH_3, and ν_s of =C-O-C, respectively, from remaining vinyl acetate units (low intensity as PVA present degree of hydrolysis > 99%).

Upon chemical crosslinking with CA (Figure S2A, PVA:CA), it was observed the increase of the band at 1714 cm^{-1} associated with νC=O vibration from citric acid, and the appearance of the vibrations at 1730 cm^{-1} (νC=O) and 1210 cm^{-1} (νC-O) associated with esterification reaction (R_2-COO-R_2, R_2 = PVA) as schematically presented in Figure S2B.
